# “I Lost My Beautiful Child”

**DOI:** 10.4269/ajtmh.21-0321

**Published:** 2021-07-06

**Authors:** Kulandaipalayam Natarajan Sindhu

**Affiliations:** Wellcome Trust Research Laboratory, Division of Gastrointestinal Sciences, Christian Medical College, Vellore, Tamilnadu, India

“What would I do, I was helpless. I lost my beautiful child. My life is a misery having married this drunkard,” the woman, who I will call Zuvaiya said. I had just met Zuvaiya moments before and was taken aback but could not agree with her more. I hadn’t known what Zuvaiya would tell me when my knuckles made their way to knock on the rusty door of Zuvaiya’s house located on a rambunctious street of Ramnaickanpalayam. As a young public health physician, facing the bereaved in the community was not uncommon. Yet, facing a mother who recently lost her child still gave me cold feet, though I knew by heart the standard lines to be spoken to the bereaved. I was there for a verbal autopsy of a 4-month-old child named Sultana,† who died recently in the semi-urban slum of Vellore.

I was already uncomfortable when I knocked on the door, and the door creaked open to reveal an elderly woman, Zuvaiya’s mother. My team nurse and I squelched along into the house, a single room really. The other side of the room ended even before I adjusted my vision in the dimly lit room. On one corner was a rickety steel cot with a ragged mattress. Diagonally flung across the room was a cramped kitchen with hackneyed aluminum vessels and disarranged plastic bottles of masalas. Yellow and green plastic pots with water stood incandescent in the windowless room filled with indomitable darkness. Zuvaiya, probably in her early twenties, barely flesh and bones, sat dismayed with her gaze fixed to the semi-concrete floor. “*Zuvaiya, doctoramma vandhirkanga, paaru*,” said her mother in Tamil [Doctor has come, see]. Zuvaiya looked at us. Her sleep-deprived eyes and premature wrinkles on her forehead like the sand dunes of a troubled dessert stood out. It was evident that she had no energy to speak, nor cry. For sure, the last few days had indeed been tumultuous to her. Tears welled up in her eyes on seeing us. A little boy (her first child) aged around 4 years sat beside her bemused, probably unable to comprehend why his baby sister was neither in his mother’s lap nor in the *thottal* (cradle). Silence perched between Zuvaiya and us over the next few, probably the longest minutes, as we sat at the edge of the bed.

Zuvaiya sat numb with her gaze again stone fixed to the ground. Her mother’s voice broke the silence and motioned for her to speak. I held her hands as she said those words that highlighted her plight: “What would I do, I was helpless. I lost my beautiful child. My life is a misery having married this drunkard.” The next few words of anguish collected from Zuvaiya revealed that her husband was a chronic alcoholic. An illiterate, he worked as a mechanic in a nearby shop. The bare minimum he earned made its way ad nauseam to the alcohol arrack shop, and rarely to the household, implying half-filled tummies many nights. Numerous days and nights witnessed him in a drunken state beating up Zuvaiya like a helpless animal.

Her story worsened one day when Sultana (her deceased 4-month-old daughter) was running a high fever for 3 days. Zuvaiya brought Sultana to the outreach clinic situated in the same lane as her home. The clinic physician immediately referred the child to the nearest secondary care hospital in view of the fast breathing and some dehydration, suspecting pneumonia. Zuvaiya returned home immediately and asked her husband to take the child to the hospital, but instead he took her forcibly to a local traditional healer (thinking hospitalization would mean he would have to arrange money and run around). The traditional healer chanted a few words in the name of god amidst Sultana and Zuvaiya and gave some native medicines. With fever unabated, Zuvaiya brought back Sultana to the outreach clinic the next day with the physician now seriously warning Zuvaiya of Sultana’s capricious clinical situation, insisting sternly that she be taken to the nearest secondary care hospital instantly. Zuvaiya, now distressed, rushed home only to find her husband drunk and passed out bungled in a corner. Penniless to take a rickshaw or even the local bus to the hospital, Zuvaiya stayed back at home ignorant of the portent she was living through that night. Sultana continued to run a high fever despite the paracetamol and the tepid sponging. Tired with exasperation, Zuvaiya fell asleep off her own guard for the next few hours beside Sultana.

She woke with a sudden fright in the wee hours of the morning and turned to immediately place the back of her palm on Sultana’s forehead. There was no fever now, but Sultana was breathing even faster. Her eyes suddenly caught the reddish stains on the pillow cover. She was confused, and now petrified. *“*Did Sultana vomit blood? Oh, my god! Please help me. I hope it is nothing serious” Immediately, she darted out of the bed to wake up her husband. “Get up! Get up! Sultana has vomited blood. Get up!”

Her husband still subsumed in deep sleep mumbled, “Go away.”

“You wretched man, get up, my child will die, take me to the hospital immediately. Allah! Help me!” screamed Zuvaiya. Zuvaiya sobbed and begged him to take Sultana to the hospital.

He got up and stormed at Zuvaiya with his blood-shot eyes and said, “*Setha saavatum, ennai veedu*” [let her die, leave me alone] and bungled back to his original state.

Zuvaiya ran out of her house not knowing what to do. She ran to her neighbor, Poonkodi’s† house and asked Poonkodi for some money. She requested Poonkodi to accompany her to the hospital. Poonkodi, seeing panic brimming Zuvaiya’s eyes, agreed to accompany her and immediately took Sultana, who was now semi-conscious, in a rickshaw to the secondary care hospital.

At the hospital emergency, Sultana was immediately assessed, and fluids were started. Blood was drawn for complete blood counts, and shortly thereafter the platelet count result read 15,000 cells/mm^3^. In view of a very low platelet count with concurrent severe pneumonia and suspected dengue hemorrhagic fever, Zuvaiya was told she must take Sultana to a higher center. Knowing her husband was in no state to do anything, she phoned her mother-in-law using Poonkodi’s mobile phone for help, only to get a blatant refusal. She decided to take Sultana to the government tertiary care hospital located a few kilometers away.

She asked the hospital for transportation, and the hospital’s ambulance immediately dropped them at the tertiary care hospital, with an accompanying junior doctor giving a quick summary of Sultana’s condition to the attending doctor. Here, an array of tests were performed, including electrolytes, blood gas, and chest X-ray. Zuvaiya was told she must take Sultana to Egmore hospital (a children’s super-specialty government hospital, about 2 hours from Vellore). Zuvaiya made numerous phone calls from Poonkodi’s phone, with her husband responding 4 hours later. Her husband finally came to the spot, very reluctantly. They were quickly transferred to Egmore by the government ambulance, and they arrived late at night. As soon as Sultana was moved to the intensive care unit in the early hours of the morning, her condition deteriorated. Sultana was declared dead by 11 AM. “The doctors fought till the last minute to save Sultana but could not,” Zuvaiya said.

Sultana’s final rites were performed back home, followed by fights between Zuvaiya and her husband every single day. Zuvaiya said that it was her husband’s negligence and his alcoholism that caused delays at multiple points when her child could have been somehow saved.

This was a situation where there was immense help available at multiple levels. Timely referrals were made, and healthcare was definitely accessible. Although there may have been a lack of insight in seeking the right healthcare point from the very beginning by Zuvaiya, it is clear that the irresponsible delays due to negligence as a father and husband catalyzed by alcoholism cost Sultana's life and furthered the misery in Zuvaiya's life.

Fifteen months later, we had an opportunity to get in touch with Zuvaiya. Zuvaiya has now separated from her husband (not legally). Her husband currently stays with another woman whom he intends to marry, and his alcoholism continues. Zuvaiya has rented a house away from Ramnaickanpalayam and has joined a shoe factory as a daily wage laborer. The money she earns hardly makes her ends meet. She however wishes to send her son to a good school to see him well educated.

Zuvaiya’s incident got me pensive and taught me something invigorating. There exists an invisible blanket of factors beyond the walls of just the provision of healthcare and access to it. The existing intricate social behaviors, domestic violence that lingers silently, recalcitrant alcoholism in men, and the lack of education for large segments of the population are larger issues that are delicately interwoven into people’s lives and loom with animosity in poor communities. Our efforts as physicians must persist to better the lives of people holistically and not merely with reference to the bodily diseases in the community. We wait with hopes for the day when no more Sultanas lose lives, nor Zuvaiyas pushed to misery.

